# Heterogeneity of memory T cells in aging

**DOI:** 10.3389/fimmu.2023.1250916

**Published:** 2023-08-18

**Authors:** Abhinav Jain, Ines Sturmlechner, Cornelia M. Weyand, Jörg J. Goronzy

**Affiliations:** ^1^ Department of Immunology, Mayo Clinic College of Medicine and Science, Rochester, MN, United States; ^2^ Department of Medicine, Division of Rheumatology, Mayo Clinic College of Medicine and Science, Rochester, MN, United States; ^3^ Robert and Arlene Kogod Center on Aging, Mayo Clinic College of Medicine and Science, Rochester, MN, United States

**Keywords:** memory T cell, aging, cytotoxic T cell, vaccine, T cell durability

## Abstract

Immune memory is a requisite and remarkable property of the immune system and is the biological foundation of the success of vaccinations in reducing morbidity from infectious diseases. Some vaccines and infections induce long-lasting protection, but immunity to other vaccines and particularly in older adults rarely persists over long time periods. Failed induction of an immune response and accelerated waning of immune memory both contribute to the immuno-compromised state of the older population. Here we review how T cell memory is influenced by age. T cell memory is maintained by a dynamic population of T cells that are heterogeneous in their kinetic parameters under homeostatic condition and their function. Durability of T cell memory can be influenced not only by the loss of a clonal progeny, but also by broader changes in the composition of functional states and transition of T cells to a dysfunctional state. Genome-wide single cell studies on total T cells have started to provide insights on the influence of age on cell heterogeneity over time. The most striking findings were a trend to progressive effector differentiation and the activation of pro-inflammatory pathways, including the emergence of CD4^+^ and CD8^+^ cytotoxic subsets. Genome-wide data on antigen-specific memory T cells are currently limited but can be expected to provide insights on how changes in T cell subset heterogeneity and transcriptome relate to durability of immune protection.

## Introduction

The adaptive immune system is able to remember previous antigen encounters due to expansion and differentiation of antigen-specific cells. This immune memory is the scientific basis for one of the most successful interventions in modern medicine, the induction of a protective immune state by vaccinations. However, immune memory is compromised in the older population. Older adults are more susceptible to infections that they have encountered earlier in life, such as rotavirus or respiratory syncytial virus. With increasing age, latent infection with the varicella zoster virus (VZV) can no longer be controlled, leading to viral reactivation presenting as shingles. By the age of 80 years, about 50% of the population has experienced at least one single episode of shingles. Moreover, vaccinations in older adults are less efficacious, with influenza being a prime example (1, 2). The mechanisms underlying lower efficacy in older individuals are incompletely understood. Defective primary responses, in part due to the reduced frequencies of naïve T cells, may explain the high morbidity and mortality with certain infections that have not been encountered at younger age such as SARS-CoV-2 or West Nile virus. However, booster vaccinations are also less effective. For example, the VZV vaccine Varivax is protective in children, while the same viral strain given in higher doses as Zostavax only provides short-term protection in older adults. The reduced efficacy may be in part due to reduced activation and expansion of antigen-specific T cells. However, this is not always the case; we have shown that the initial expansion of antigen-specific T cells after Zostavax vaccination is independent of age, while the contraction in frequencies after peak responses is more severe in older adults, likely due to increasing DNA damage and replicative stress responses (3, 4). We have recently reviewed the mechanisms underlying this impaired generation of long-lived memory T cells in older adults (5). Equally important is the question how long memory T cells live and how well their functionality is maintained into older age. Here, we review whether and how memory T cells change with increasing age, focusing primarily on data from human studies.

### Durability of T cell memory

Clinical data on immune protection document that immunity can be remarkably long-lived. Longitudinal studies after vaccination are especially informative. A single dose of the yellow fever vaccine (YFV) that causes a short infection lasting about one week confers life-long immunity (6). Similarly, an influenza strain circulating shortly after 1918 induced immunity that protected the very elderly from the 2009 H1N1 swine flu influenza epidemic (7, 8). These examples demonstrate the power of immune memory over lifetime when the virus was first encountered in children or young adults. However, this degree of memory durability is certainly not generalizable to immune memory generation in older adults. A typical example of vaccines with age-dependent protection span is the live-attenuated vaccine against VZV. Protection against VZV is thought the be predominantly conferred by T cells (9, 10). The childhood vaccine, Varivax, and the adulthood vaccine for 50+ year-olds, Zostavax, are based on the same vaccine formulation, albeit Zostavax containing ~14 times higher particle titers. Despite that the vaccine is virtually identical, the protection rates differ drastically. Vaccination of children allows for long-lasting protection from VZV infection of 10+ years (11–13). In contrast, vaccination of older adults aged 50-70 years prompts a short-term immune memory with shingles protection rates waning within four years post-vaccination in most elderly (14, 15). Age-related changes, including T cell-intrinsic molecular maladaptations that may skew T cell protection to wane faster, are incompletely understood. Still, targeted design of vaccines to the older population is feasible and clinically highly relevant, as demonstrated by the adjuvanted VZV subunit vaccine, Shingrix. Shingrix vaccination confers high protection rates against shingles in 50+ year-old adults that, based on prediction models, is expected to last up to 19 years (16).

### Immune memory is maintained by a dynamic population

**Figure 1:**
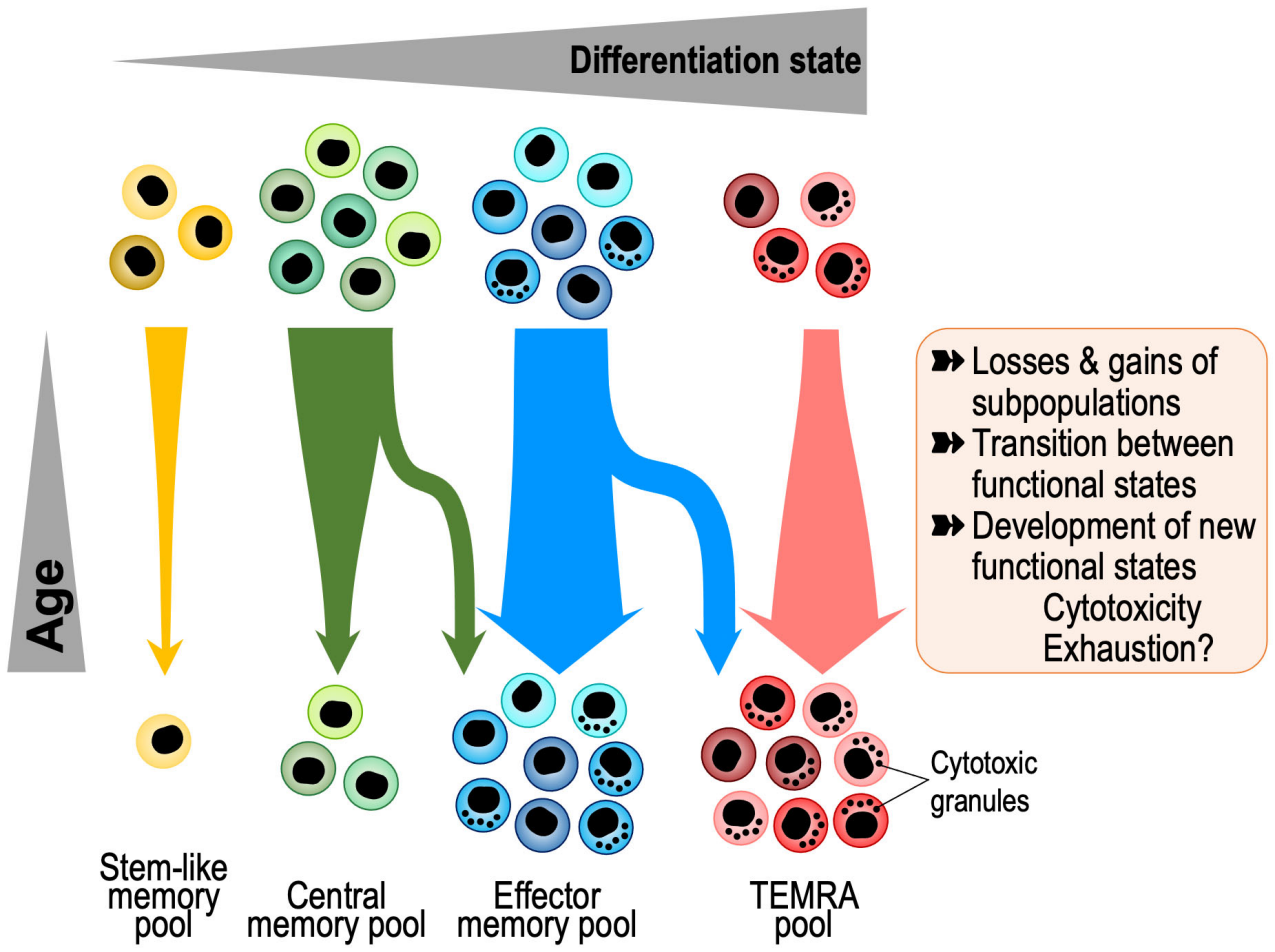
Age-associated changes in memory T cell composition with aging. Memory T cells are a heterogeneous and dynamic population that undergoes several kinds of transitions, including loss or gains of subpopulations and changes in gene expression. Recent insights into these transitions come from single cell studies that show shifts towards progressive differentiation and effector functions, including gain in cytotoxicity and exhaustion states and loss of stem-like and central memory cells. TEMRA, Terminally Differentiated Effector Memory Cells with CD45RA expression.

In a simplified model, longer protection may just reflect the higher survival of individual antigen-specific memory cells. Indeed, individual T cell progenies can be very long-lived. In studying the T cell receptor repertoire of older individuals, we have identified T cells with identical amino acid sequences in identical twins but not in unrelated adults (17). These sequences were more frequently identical than expected at the nucleotide sequence, indicating that they derived from the same progenitor cell that must have been seeded *in utero* at a time when the fetal circulations were connected. However, it is important to note that long-term memory is maintained by a dynamic population that is in constant turn-over. T cell memory is conferred by a population of cells that individually are more short-lived, certainly considerably shorter than the duration of immunological memory (18). The lifespan of a human memory T cell is 30-160 days (19–22), in contrast to the typical half-life of human T cell memory of 8-15 years (6, 23). Akondy et al. directly determined the turnover rate of YFV-specific CD8^+^ T cells around day 42 and day 365 after vaccination (24). Assuming constant rates for both the loss in cell numbers and the turnover of these cells, the estimated average rates for cell loss and rate of division of YFV-specific cells during this period were 0.57 ± 0.08% per day (half-life of about 122 days) and 0.15 ± 0.09% per day (cells divide on average once every 462 days), respectively. Zarnitsyna et al. modelled these and additional frequency data from Fuertes Marraco et al. (25) and concluded that the kinetics followed a power law rather than an exponential or bi-exponential model (26). Decay rates declined over time, while division rates did not asymptote to zero supporting the notion that long-term memory in humans is maintained by a population undergoing turnover. Longevity is therefore determined by the division rate as well as the rate of loss and cell death. Memory reflects population averaging of a very diverse set of cells, rather than uniformity at the single cell level. Whether and how these kinetic parameters change with age is currently unknown. In addition to division and death rates, transition rates between different functional states contribute to change in diversity and memory. Taken together, loss of a clonal progeny, change in the composition of functional states or transition to a dysfunctional state such as senescence or exhaustion could all result in a decline of immune memory with older age (**Figure 1**).

### Increased vulnerability of CD8^+^ T cells to age-associated changes

Early flow cytometric studies on the influence of age on T cell heterogeneity documented that CD8^+^ T cells are much more susceptible to undergo phenotypic transitions than CD4^+^ T cells. While both naïve CD4^+^ and CD8^+^ T cells decline with age, the magnitude of this decline is strikingly higher for CD8^+^ T cells (27). In fact, in a large population study, loss of naïve CD8^+^ T cells was the strongest immunological correlate of age while loss of naïve CD4^+^ T cells was less significant (28). The lineage-associated difference extends to central memory and effector memory T cells. The CD8^+^ compartment of central memory T cells is small and further declines with age. Most stem-like memory CD8^+^ T cells are included in the phenotypically defined naïve CD8^+^ T cell compartment (25, 29), while stem-like memory CD4^+^ T cells are part of the central memory compartment (30) that is more stable over lifetime. Expansion of effector memory and particularly terminally differentiated effector (TEMRA) cells is common for CD8^+^ T cells and driven by age and chronic infection. High frequencies of CD4^+^ TEMRA are less common but are found in supercentenarians making up 15-35% of total CD4^+^ T cells (31). These data suggest that expansion of both CD4^+^ and CD8^+^ cytotoxic T cells with age is a common age-related signature.

However, CD4^+^ T cells are protected from end-differentiation for many years. One underlying mechanism for CD4^+^ T cells to eventually lose this state of youthfulness and convert to TEMRA with older age is the loss of homeostatic quiescence involving the lineage-determining transcription factors ThPOK and RUNX3 that regulate the expression of TRIB2 (32). A subset of CD4^+^ T cells lose ThPOK and TRIB2, while gaining RUNX3 expression with age. This allows for increased AKT signaling, expression of low CD8 levels in CD4^+^ T cells and acquisition of a TEMRA phenotype, suggesting a tendency of both memory T cell lineages to converge with age while acquiring cytotoxic and NK-like features.

### Age-associated epigenetic signatures resemble progressive differentiation

Age-associated phenotypic changes on a population level are more apparent in flow cytometry studies for CD8^+^ memory T cells, frequently due to the emergence of clonally expanded populations that express CD57 or produce granzyme B. The emergence of genome-wide technologies enabled more granular studies. An initial analysis of age- and *cis*-gene expression-associated methylation sites (age-eMS), integrating genome-wide CpG methylation and gene expression profiles collected from circulating CD4^+^ T cells identified 227 sites, none of which were shared with monocytes (33). Age-eMS tended to be hypomethylated with older age, located in predicted enhancers. However, this study examined global CD4^+^ T cells without accounting for differentiation stages. Subsequent epigenetic studies mapping chromatin accessibility in T cells stratified for CD4^+^ and CD8^+^ naïve, central and effector memory cells identified differentially accessible sites that included motifs for transcription factors associated with effector cell differentiation (34). CD8^+^ T cell subsets were more affected than CD4^+^ T cells (35, 36). These changes were observed although TEMRAs were excluded in these studies and study participants were selected to be heathy, non-frail older adults who are generally less immunocompromised. Consistent with the resilience of CD4^+^ T cells, transcriptional profiling of circulating Tfh cells from young and older individuals before and after influenza vaccination showed expected signatures for functional Tfh cells (37). Expression of individual genes did not reveal obvious age-related changes that could indicate dysfunction. Pathway analysis, however, showed higher enrichment for IL-2/STAT5 and TNF/NF-κB signaling in ICOS^+^CD38^+^ conventional Tfh from older adults on day 7 after vaccination. Although not proven by the authors, STAT5 signaling could induce BLIMP1 expression that would render Tfh cells dysfunctional by inhibiting BCL6 (38). The age-associated activation of these pathways appears to be a general feature of T cell aging and not limited to Tfh cells. Bektas et al. reported a low-level activation of the PI3K/NF-κB pathway in T cells from older adults (39). Zhang et al. identified activation of STAT5 early in naïve CD4^+^ T cell responses from older adults as the major cause of accelerated chromatin remodeling and effector cell differentiation (40). Moreover, expression of CD39 is more rapidly induced in memory T cells from older adults (41). CD39 is an ecto-ATPase expressed on activated effector cells, including exhausted CD8^+^ T cells, and has been implicated in cell death and immunosuppression through the production of adenosine. Its transcription is repressed by BCL6 while induced by BLIMP1 and RUNX3, transcription factors involved in effector cell differentiation. Taken together, the epigenetic landscape in T cells from older adults is poised towards expression of effector cell genes that may contribute to increased tissue invasion and the subtle systemic inflammation characteristic of older adults. These traits are in line with the concept of inflammaging, the age-related accumulation of stressors that result in a sterile, chronic, low-level pro-inflammatory state in the organism (42). This pro-inflammatory, epigenetic signature contrasts to that in stem-like memory T cells and may therefore have a negative impact on immune memory durability.

### Influence of age on memory cell heterogeneity

Single cell (sc) sequencing has revolutionized the field of immunology making it exceptionally suited to distinguish the heterogeneity of immune cell subsets at transcriptional, epigenetic, translational, spatial, mutational, as well as TCR (T cell receptor), and BCR (B cell receptor) levels. Integration of these modalities provides in-depth information of the changes in immune cells with aging as well as the identification of rare/novel cell types (43) (**Figure 1**). In addition, one can identify the cellular and functional characteristics of immune cell types and their variability between individuals and tissue during aging. Currently, there are 11 published single cell (scRNA, scATAC, scCITE, scTCR) studies focused on T cell aging including 4 on only CD4^+^ T cells, 2 on only CD8^+^ T cells and 5 on all T cells, involving seven studies in humans, three in mice and one in humans and mice (31, 40, 44–52). Overall, with aging, the transcriptome of immune cells shifts towards the expression of inflammation-related genes (48). The most common age-associated changes identified in CD8^+^ T cells are shifts to more differentiated cells, i.e., effector, TEMRA, and T cells expressing exhaustion markers. Also, there is increased clonality of effector, cytotoxic, and exhausted CD8^+^ T cells and decreased diversity and complexity of the TCR repertoire with age (45, 47, 53). Interestingly, exhausted T cells have high expression of the lncRNA *NEAT1* and *MALAT1*. An infrequent population of CD8^+^ T cells that increases with age are GZMK-expressing, age-associated T cells (Taa) (51). These Taa cells express PD-1 and TOX in the mouse indicating exhaustion, but not in humans, where they express EOMES consistent with functional effector memory cells. They accumulate in multiple tissues and secrete GZMK that in turn predisposes mesenchymal cells to become senescent. Using CD8^+^ T cell single cell transcriptome data, Lu et al. developed a linear model predicting the biological age that they correlated with the mutational burden of each immune subset (naive, EM, CM, TEMRA). The highest increase in mutational burden with age was in the TEMRA population (45).

For CD4^+^ memory T cells, Elyahu et al. identified a gain in cytotoxicity as the major age-associated change (49). The function of cytotoxic CD4^+^ T cells is incompletely understood. These cells are also generated against herpes viruses such as human herpes virus 6B (HHV-6B) and have high polyfunctionality (54). Cytotoxic CD4^+^ T cells can contribute to host defense against lethal influenza virus infection in mice (55) and they can provide anti-tumor responses (56). In a recent study, skin resident cytotoxic CD4^+^ T cells were shown to also eliminate senescent cells that are implicated in driving many aspects of aging (57). Senescent skin fibroblasts presented peptides of CMV glycoprotein B via MHC class II making them sensitive to cytotoxic CD4^+^ T cell-mediated killing. In humans, cytotoxic CD4^+^ T cells are clonally expanded in supercentenarians, suggesting that they are beneficial for healthy aging (31). Conversely, cytotoxicity of CD4^+^ T cells in the context of MHC class II molecules which are mostly expressed on antigen-presenting cells could also entail a detrimental effect by regulating the frequencies of dendritic cells. Such an inhibitory effect could manifest in peripheral tissue but also in lymph nodes. Previous studies employing adoptive transfer of cytotoxic CD4^+^ T cells have suggested that these cells can home not only to peripheral tissues but also to lymph nodes (58). scRNA-seq studies of CD4^+^ T cells after activation identified increased cell-to-cell variability in old mice compared to the tight regulation in young mice (44).

So far, single cell studies have highlighted the heterogeneity of the memory T cell compartment and gain in cytotoxic function, while evidence for senescence or exhaustion is not definite and the mechanisms causing dysfunction have remained elusive. However, it should be noted that studies on antigen-specific T cells are needed to interpret age-associated shifts. Global population studies such as the ones referenced above do not allow drawing conclusion on the driving forces, e.g., whether shifts truly reflect aging or whether they are the result from latent infections. Interestingly, a recent mouse study indicated that the memory T cell pool can avoid age-associated dysfunction. Serial adoptive transfers of antigen-specific T cells allowed the authors to monitor antigen-specific memory T cells far beyond the normal lifespan of a mouse (59). The study suggested that the memory T cell pool survives repeated antigen-stimulation without displaying evidence of impaired clonal expansion or telomere erosion. Moreover, these cells can avoid terminal exhaustion and cellular senescence depending on the interval between stimulations. Human studies controlling for antigen-specificity by comparing tetramer-sorted T cells of young and older adults have not been published.

### Negative regulatory receptors - not necessarily a marker of exhaustion

**Figure 2:**
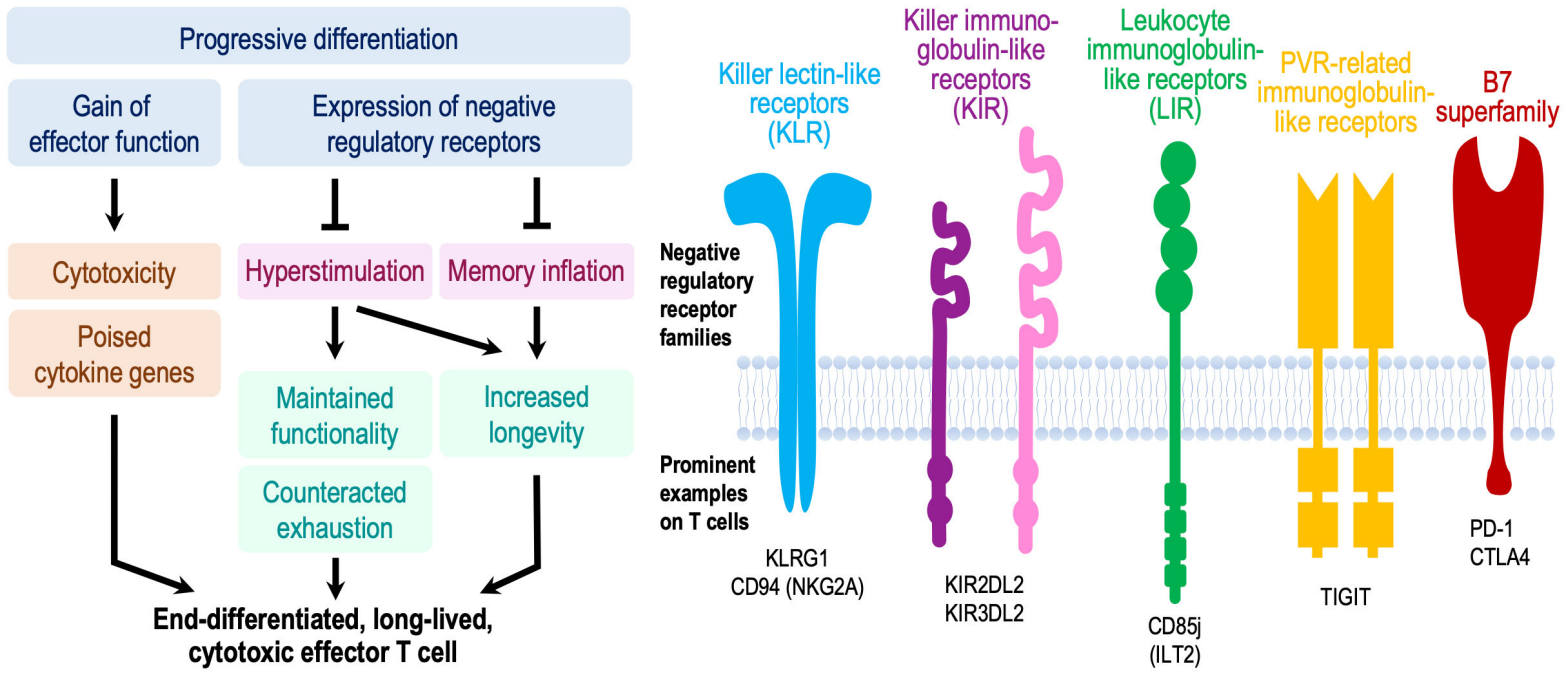
Model proposing a beneficial effect of negative regulatory receptors for T memory cell longevity and function. Concomitant to gaining cytotoxic function, CD4^+^ and CD8^+^ effector memory cells in older adults can express a multitude of negative regulatory receptors that are regularly expressed on NK cells or exhausted CD8^+^ T cells. While these receptors attenuate proximal signaling, these cells are functional and poised to exhibit effector functions. The current paradigm implicates these receptors as a means of controlling memory inflation. We propose that their major importance lies in preventing chronic hyperstimulation and thereby improving immune memory durability and long-term maintenance of functional competence including regulated cytokine production and cytotoxicity. This model is reminiscent to functional licensing by inhibitory KIR, KLR and LIR receptors in NK cells, or to prevention of terminal exhaustion in T_ex_ precursor cells. Consistent with this model, cytotoxic T cells accumulate in supercentenarians.

The major transcriptional and epigenetic signature associated with T cell aging in genome-wide studies is the activation of cytotoxic genes as described above. Single cell studies have linked these signatures to inhibitory receptors that are expressed on exhausted CD8^+^ T cells, such as PD-1, LAG-3, and TIM-3. Whether these cytotoxic cells are terminally exhausted, i.e., whether transcriptional activation of effector genes is impaired in addition to the signaling inhibition by these receptors (60), has remained unclear. The finding of inhibitory receptors on memory T cells due to progressive differentiation and older age is not limited to exhaustion-associated molecules and their functional implications has been a point of controversal discussion. Phenotypic studies already two decades ago provided evidence that the subsets of CD8^+^ and CD4^+^ T cells that had gained cytotoxic properties expressed inhibitory receptors (**Figure 2**) (61–64). Such receptors included MHC class I-specific members of the killer immunoglobulin-like receptor (KIR) and killer cell lectin-like receptor (KLR) families, in particular KLRG1, and CD85j of the leukocyte immunoglobulin-like receptor (LIR) family. The age-associated expression of these receptors appeared to be related to a progressive promoter demethylation due to reduced local DNMT1 activity as also seen for cytotoxic genes, tying together the transcriptional activation of these gene groups (65). T cells expressing these receptors are oligoclonally expanded (66, 67) and include herpes virus-specific T cells (61, 68). Similar to their function in NK cells, MHC class I-specific inhibitory receptors were thought to limit the clonal expansion and prevent memory cell inflation (69). However, several studies have shown that this is not necessarily the case, and these cells can remain functional effector cells. For example, recruitment of the inhibitory receptor KIR2DL2 inhibited sustained TCR signaling and reduced transcription of cytokines but did not affect cytotoxicity (70). Also, virus-specific CD8^+^ T cells expressing CD85j retained their full cytotoxic potential when CD85j was blocked. Although their proliferative potential and cytokine production may be slightly heightened with CD85j blockade (68, 71), these studies demonstrate that cytotoxic T cells expressing negative regulatory receptors are still highly functional and not epigenetically or terminally dysfunctional. Moreover, cytotoxicity in TEMRA or the previously described Taa cells is linked to the expression of the transcription factor EOMES even if they express PD-1 (51). Therefore, they are more similar to fully functional precursor cells than terminally exhausted cells (72). It has been proposed that the expression of PD-1 on TCF1-expressing precursor exhausted cells in the presence of abundant antigen prevents hyperstimulation and the epigenetic changes that confer functional and terminal exhaustion (72). In that sense, the expression of negative regulatory cell surface receptors on cytotoxic cells could be protective and promote the longevity of these memory T cells (**Figure 2**). This mechanism differs from stem-like memory cells that gain longevity by reverting to a quiescent state with high TCF1 expression (73). High longevity of cytotoxic T cells would be consistent with the observation that they accumulate in centenarians (31).

## Concluding remarks

T cell memory is maintained by a dynamic cell population that includes cells with very different kinetics such as relatively quiescent stem-like memory cells and terminally differentiated TEMRA cells. Data on whether and how the population kinetics is changing with age would be important to understand and improve the durability of immune memory after vaccination. Such data are lacking, at least at the level of antigen-specific cells. Alternatively, studies of memory T cell heterogeneity and its changes over lifetime can be informative. Single cell transcriptome and epigenome studies have provided information on global population shifts but we still do not know much on changes in population compositions of antigen-specific T cells. For example, we assume that the reduced expression of TCF1 with age may negatively impact the survival of stem-like memory cells, but data are lacking. Conversely, we know that end-differentiated effector cells accumulate with age, likely as a result of frequent restimulation. While these cells express negative regulatory receptors, they do not appear to be exhausted, and the inhibitory receptors may actually be beneficial for maintaining their functionality. Taken together, our knowledge on why immune memory declines with age remains limited. Conversely, we have strong evidence that aging is associated with progressive differentiation of T cells towards effector phenotypes with activation of inflammatory and cytotoxic pathways and gain in tissue invasiveness. This age-associated differentiation is more evident for CD8^+^ than CD4^+^ T cells due to the difference in lineage-specific gene-regulatory factors. The pro-inflammatory poised state appears to be gained due to activation of T cell-inherent regular differentiation program rather than cellular senescence, although the inflammatory mediators are overlapping. Nonetheless, this propensity is likely an important contributor to the subtle, systemic inflammatory statue of older adults.

## Author contributions

AJ, IS, CW, and JG wrote and edited the review. All authors contributed to the article and approved the submitted version.
